# Multimodal Predictions of Super-Refractory Status Epilepticus and Outcome in Status Epilepticus Due to Acute Encephalitis

**DOI:** 10.3389/fneur.2018.00832

**Published:** 2018-10-08

**Authors:** Fang Yuan, Fang Yang, Ruihua Jia, Wen Li, Yongli Jiang, Jingjing Zhao, Wen Jiang

**Affiliations:** Department of Neurology, Xijing Hospital, Fourth Military Medical University, Xi'an, China

**Keywords:** status epilepticus, encephalitis, super-refractory status epilepticus, neuroimaging, continuous electroencephalogram, multimodal prediction

## Abstract

**Objective:** Status epilepticus (SE) is one of the most critical symptoms of encephalitis. Studies on early predictions of progression to super-refractory status epilepticus (SRSE) and poor outcome in SE due to acute encephalitis are scarce. We aimed to investigate the values of neuroimaging and continuous electroencephalogram (EEG) in the multimodal prediction.

**Methods:** Consecutive patients with convulsive SE due to acute encephalitis were included in this study. Demographics, clinical features, neuro-imaging characteristics, medical interventions, and anti-epileptic treatment responses were collected. All the patients had EEG monitoring for at least 24 h. We determined the early predictors of SRSE and prognostic factors of 3-month outcome using multivariate logistic regression analyses.

**Results:** From March 2008 to February 2018, 570 patients with acute encephalitis were admitted to neurological intensive care unit (N-ICU) of Xijing hospital. Among them, a total of 94 patients with SE were included in this study. The percentage of non-SRSE and SRSE were 76.6 and 23.4%. Cortical or hippocampal abnormality on neuroimaging (*p* = 0.002, OR 20.55, 95% CI 3.16–133.46) and END-IT score (*p* < 0.001, OR 4.07, 95% CI 1.91–8.67) were independent predictors of the progression to SRSE. At 3 months after N-ICU discharge, 56 (59.6%) patients attained good outcomes, and 38 (40.4%) patients had poor outcomes. The recurrence of clinical or EEG seizures within 2 h after the infusion rate of a single anesthetic drug >50% proposed maximal dose (*p* = 0.044, OR 4.52, 95% CI 1.04–19.68), tracheal intubation (*p* = 0.011, OR 4.99, 95% CI 1.37–11.69) and emergency resuscitation (*p* = 0.040, OR 9.80, 95% 1.11–86.47) predicted poor functional outcome.

**Interpretation:** Initial neuro-imaging findings assist early identification of the progression to SRSE. Continuous EEG monitoring contributes to outcome prediction in SE due to acute encephalitis.

## Introduction

Encephalitis is an inflammatory process of the brain, with an incidence of 3.5–12.6 cases per 100,000 patient-years worldwide (1, 2). Patients with acute encephalitis typically present with acute onset of fever, impaired consciousness, headache, seizures, or new onset of focal neurologic deficits (3). Acute encephalitis is a severe form of neurological illnesses that usually requires intensive care for monitoring and treatment. Reported mortality rates range between 7 and 18%, and up to 56% of survivors suffer from severe disability (4–7).

Status epilepticus (SE) is one of the most common neurological symptoms of encephalitis, occurring in 18.5% cases of acute encephalitis (5). Previous studies suggested that younger age, coma, cortical lesions on neuroimaging, and nonneurologic organ failure were risk factors for the incidence of SE in patients with encephalitis (8, 9). SE due to acute encephalitis is a critical condition that is strongly associated with higher refractoriness (10, 11). It often evolves to super-refractory status epilepticus (SRSE) and consequently results in higher mortality (5, 10, 11). Early identifications of the patients with higher risks of progression to SRSE and poor outcomes will help clinicians orient treatment strategies and may improve the outcomes of SE in acute encephalitis.

Given the current paucity of studies regarding the aforementioned problems, we conducted a 10-year retrospective study in the neurological intensive care unit (N-ICU) to investigate the contributions of brain magnetic resonance imaging (MRI) and electroencephalogram (EEG) monitoring in the multimodal predictions of progression to SRSE and 3-month poor outcome in SE due to acute encephalitis.

## Materials and methods

### Design and setting

This study was based on a prospective database of acute encephalitis patients in N-ICU at Xijing hospital, China, a tertiary academic hospital. It was registered in ClinicalTrials.gov (NCT02278016) and approved by the ethics committee of the Xijing Hospital (KY20140916-3). We adhered to Chinese laws and the Declaration of Helsinki.

### Patients

From March 2008 to February 2018, all consecutive patients with convulsive SE due to acute encephalitis and aged 13 years or older were included in this study. Acute encephalitis was defined as encephalopathy (altered mental status lasting ≥24 h with no alternative cause identified), and three or more of the following: documented fever ≥38°C within the 72 h before or after presentation; generalized or partial seizures not fully attributable to a preexisting seizure disorder; new onset of focal neurologic findings; CSF WBC count ≥5/cubic mm; abnormality of brain parenchyma on neuroimaging (suggestive of encephalitis); abnormal electroencephalogram (EEG) findings (consistent with encephalitis) (3). According to the operational definition proposed by International League Against Epilepsy, we defined convulsive SE as 5 min or more of continuous motor seizure activity or recurrent seizure activity without regaining full consciousness between episodes (12).

### Management

The management of SE adhered to related guidelines (13–15). Benzodiazepines were administered as the first-line agents, followed by intravenous sodium valproate or phenobarbital sodium to treat persisting SE. In patients who were resistant to both first-line and second-line agents, midazolam or propofol was administered continuously as the third-line treatment. The initial loading dose of midazolam was 0.2 mg/kg, and the proposed maximal dose (PMD) of maintenance infusion rate for midazolam was 0.4 mg/kg/h (14, 15). The initial loading dose of propofol was 2 mg/kg, and the PMD of maintenance infusion rate of propofol was 10 mg/kg/h (14, 15). When a single anesthetic with PMD failed to control SE, simultaneous polytherapy of continuous infusion of anesthetics (CIVADs) was administered (16–19). All the SE patients received bedside video-EEG monitoring for at least 24 h with an array of 20 scalp electrodes (Solar 2000 N, Solar Electronic Technologies Co., Ltd., Beijing, China) to guide anti-seizure treatments and detect non-convulsive epileptic seizures.

### Data collection

The following measures were recorded and assessed: (1) variables before N-ICU admission including time from onset of encephalitis to N-ICU admission, time from onset of encephalitis until diagnosis of SE, seizures before admission, and history of epilepsy; (2) severity of illness including Glasgow Coma Scale (GCS), Status Epilepticus Severity Score (STESS) (20), and END-IT score (11); (3) encephalitis etiology diagnosed according to related guidelines and consensuses (21–26); (4) complication of non-convulsive status epilepticus (NCSE) in coma; (5) brain image (abnormal brain MRI findings were defined as hypointensity on T1WI and hyperintensity on T2WI and FLAIR); (6) N-ICU managements including length of EEG monitoring, number of intravenous antiepileptic drugs (IV AEDs), use of CIVADs, CIVAD > 50% PMD, CIVADs changed, immune therapies (including steroids, immunoglobulins, plasma exchange, and rituximab), tracheal intubation, use of vasopressors, and emergency resuscitation; (7) antiepileptic treatment responses including clinical or EEG seizures within 2 h after CIVAD, clinical or EEG seizures within 2 h after CIVAD >50% PMD, breakthrough seizures, and withdrawal seizures. NCSE in coma was defined as a type of SE, which happened in comatose patients, without motor movements or with manifestations of continuous and rhythmic phenomenon of more subtle motor twitches of the eyelid, jaw, face, trunk or extremities (12, 16). Emergency resuscitation was defined as administering emergency measures to sustain the vital functions of a person in severe respiratory and circulatory failure, malignant arrhythmia, or cardiac arrest. CIVAD was changed when a second CIVAD (monotherapy) was used because of the poor seizure control. Breakthrough seizures were defined as any clinical or EEG seizures occurring after the first 6 h of the initial CIVAD treatment; withdrawal seizures were defined as any clinical or EEG seizures occurring within 48 h after initially discontinuing or tapering the CIVAD (27, 28). Clinical seizures were defined as any epileptic seizures with perceivable motor movements. EEG seizures were defined as any spikes, sharp waves, or sharp and slow wave complexes lasting for ≥10 s at either a frequency of at least three per second or a frequency of at least one per second with clear evolution in frequency, morphology, or location (28, 29).

### Outcomes

Refractory status epilepticus (RSE) was defined as SE that continued despite treatment with benzodiazepines and one antiepileptic drug (30). SRSE was defined as SE that continued or recurred 24 h or more after the onset of anesthetic therapy (31). Three-month functional outcome was assessed via telephone interviews by a trained study assistant using Modified Rankin Scale (mRS), who was blind to the clinical data. A mRS >3 (severe disability and death) was considered as poor outcome, and a mRS ≤ 3 (normal, slight and moderate disability) was considered favorable outcome.

### Statistics

Univariate comparisons of categorical variables were performed using χ^2^-test analysis. For continuous variables, normal and non-normal distributions were distinguished by the Shapiro-Wilk test. The comparisons of normally distributed variables were performed using the Student *t*-test, and the comparisons of non-normally distributed variables were performed using the Mann-Whitney *U*-test. Age, gender, and potential risk factors with a significance level <0.05 in the univariate comparisons were included into univariate and multivariate (stepwise backward) logistic regression analyses to examine their associations with a certain outcome by estimating odds ratios (ORs) and associated confidence intervals (CIs). Two-sided *p* ≦ 0.05 were considered significant. Statistical analysis was performed with SPSS version 22 software (SPSS Inc., Chicago, IL, United States).

## Results

### Demographics and clinical features

**Figure 1:**
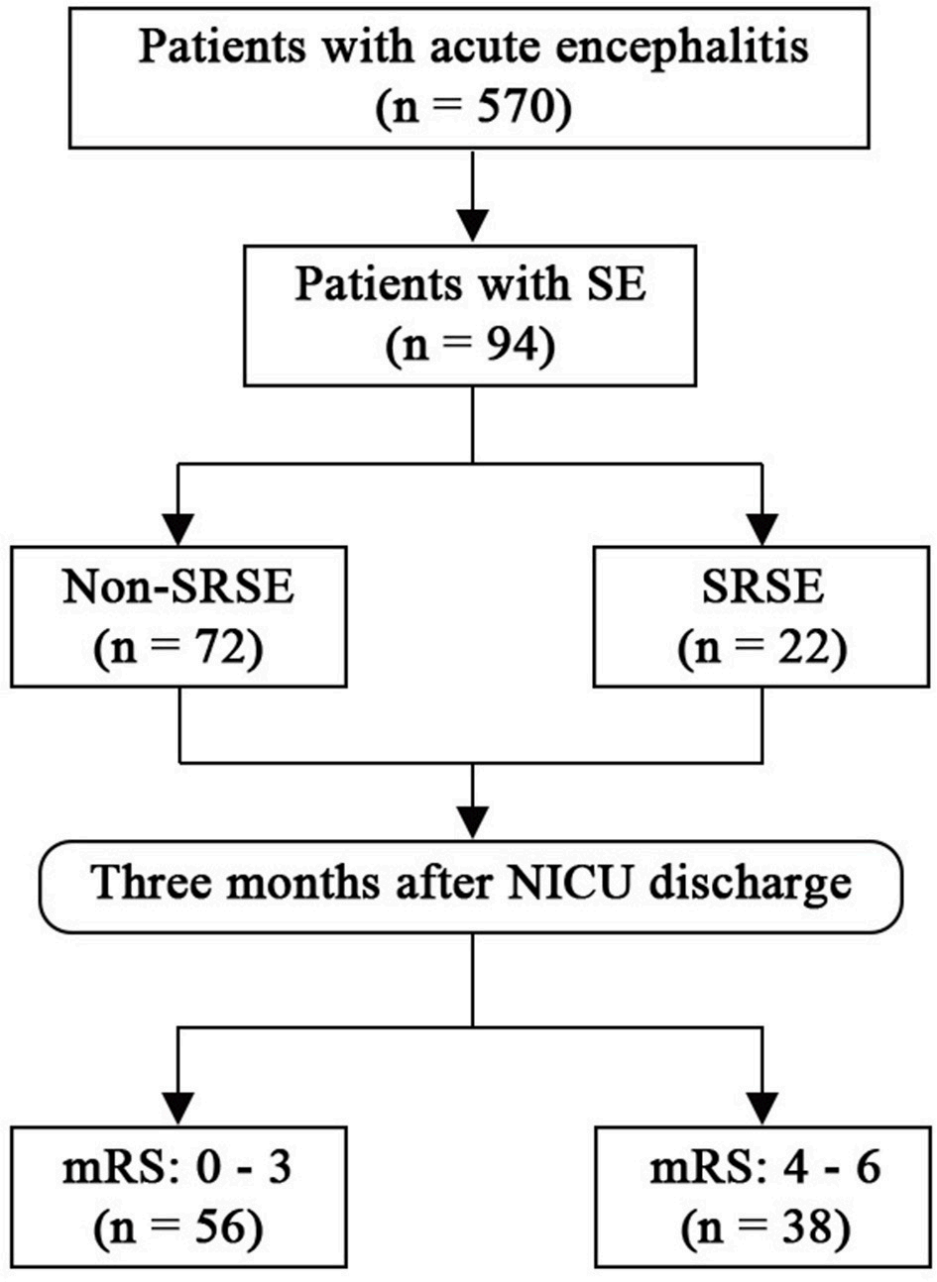
Flow chart. mRS, modified Rankin Scale; SE, status epilepticus; SRSE, super-refractory status epilepticus.

Between March 2008 and February 2018, 570 patients with acute encephalitis were admitted to N-ICU (Figure 1). Among them, a total of 94 patients with SE were included in this study. The median age of the study cohort was 26 years old (Table 1), and 55 (58.5%) patients were male. The median time from onset to SE was 5 days, and the median time from onset to N-ICU admission was 11 days. Eighty-nine (94.7%) patients had seizures before admission, and only eight (8.5%) patients had a history of epilepsy. Most patients had unknown causes (42.6%), followed by viral encephalitis (28.7%), autoimmune (22.3%), bacterial (4.3%), cryptococcosis (1.1%), and neurosyphilis (1.1%).

**Table 1:** Demographics and clinical characteristics of patients with status epilepticus associated with acute encephalitis.

	**Total (*n* = 94)**	**Non-SRSE (*n* = 72)**	**SRSE (*n* = 22)**	***P* value**
Age, year	26 (18–42)	32 (19–45)	22 (15–30)	0.017
Male (%)	55 (58.5)	44 (61.1)	11 (50.0)	0.355
Time from onset to NICU admission, day	11 (7–22)	10 (5–21)	13 (8–24)	0.264
Time from onset to SE, day	5 (3–11)	7 (3–14)	5 (3–7)	0.290
Seizures before admission (%)	89 (94.7)	67 (93.1)	22 (100.0)	0.204
History of epilepsy (%)	8 (8.5)	7 (9.7)	1 (4.5%)	0.446
GCS	9 (6–12)	9 (6–12)	6 (3–10)	0.006
STESS	4 (3–5)	4 (3–4)	5 (4–5)	<0.001
END-IT score	3 (3–4)	3 (2–3)	3 (3–4)	<0.001
Encephalitis etiology (%)				0.512
Viral	27 (28.7)	22 (30.6)	5 (22.7)	
Bacterial	4 (4.3)	4 (5.6)	0 (0.0)	
Cryptococcosis	1 (1.1)	1 (1.4)	0 (0.0)	
Neurosyphilis	1 (1.1)	1 (1.4)	0 (0.0)	
Autoimmune	21 (22.3)	17 (23.6)	4 (18.2)	
Unknown	40 (42.6)	27 (37.5)	13 (59.1)	
NCSE in coma (%)	53 (56.4)	33 (45.8)	20 (90.9)	<0.001
Brain image (%)				0.034
Normal	34 (36.2)	25 (34.7)	9 (40.9)	
Cortical or hippocampal involvement	27 (28.7)	17 (23.6)	10 (45.5)	
Exclusively abnormalities in other areas^*^	33 (35.1)	30 (41.7)	3 (13.6)	

*GCS, Glasgow coma scale; NCSE, nonconvulsive status epilepticus; NICU, neurological intensive care unit; SRSE, super-refractory status epilepticus; STESS, status epilepticus severity score*.

* *Brain parenchyma except cortex and hippocampus*.

*Data presented as n (%) or median (interquartile range)*.

### Early predictors for progression to SRSE

Forty-one (43.6%) patients with SE due to acute encephalitis evolved into RSE, and 22 (23.4%) patients evolved into SRSE. Patients with SRSE had significantly lower GCS score (*p* = 0.006), higher STESS (*p* < 0.001) and END-IT score (*p* < 0.001; Table 1). There were significantly more patients with MRI abnormalities on the cortex or hippocampus in SRSE group (*p* = 0.034). Results from multivariate logistic regression analysis (Table 2) showed that END-IT score (*p* < 0.001) and cortical/hippocampal abnormality on MRI (*p* = 0.002) were independent predictors for progression to SRSE. The cut-off point of 4 in END-IT score produced the optimal sum of sensitivity and specificity for the prediction of the progression to SRSE.

**Table 2:** Logistic regression analysis for predictors of SRSE in acute encephalitis.

**Variables**	**Unadjusted analysis**			Adjusted analysis^**^		
	**OR**	**95% CI**	***p*-value**	**OR**	**95% CI**	***p*-value**
Age	0.96	0.92–0.99	0.021			
Male	0.64	0.24–1.66	0.356			
GCS	0.83	0.72–0.96	0.011			
STESS	5.36	2.19–13.14	<0.001			
END-IT score	2.36	1.45–3.86	0.001	4.07	1.91–8.67	<0.001
NCSE in coma	11.82	2.57–54.34	0.002			
Brain image			0.050			0.007
Cortical or hippocampal involment	5.88	1.42–24.35	0.015	20.55	3.16–133.46	0.002
Normal	3.60	0.88–14.75	0.075	4.30	0.87–21.33	0.074
Exclusively abnormalities in other areas^*^	1.00			1.00		

*GCS, Glasgow coma scale; NCSE, nonconvulsive status epilepticus; SRSE, super-refractory status epilepticus; STESS, status epilepticus severity score*.

* *Brain parenchyma except cortex and hippocampus*.

** *Hosmer and Lemeshow Test: p = 0.935*.

### NICU management

Table 3 showed the managements in N-ICU for all the patients with SE due to acute encephalitis, including RSE and SRSE cases. The length of EEG monitoring for the whole cohort was 46 (28–81) hours, for RSE cases was 77 (47–171) hours, and for SRSE cases was 105 (69–274) hours. Forty-five (47.9%) patients with SE due to acute encephalitis received CIVADs, and 22 (23.4%) patients received CIVADs with more than 50% PMD. Immune therapies were used in 25 (26.6%) cases, tracheal intubation was used in 51 (54.3%) cases, vasopressors were used in 32 (34.0%) cases, and emergency resuscitation was used in 12 (12.8%) cases.

**Table 3:** NICU management and treatment responses of status epilepticus in acute encephalitis.

	**SE (*n* = 94)**	RSE^*^ (*n* = 41)	**SRSE (*n* = 22)**
Length of EEG monitoring, h	46 (28–81)	77 (47–171)	105 (69–274)
Number of IV AEDs	2 (1–3)	3 (3–4)	4 (3–4)
Use of CIVADs (%)	45 (47.9)	41 (100.0)	22 (100.0)
CIVAD >50% PMD (%)	22 (23.4)	22 (53.7)	18 (81.8)
Seizures within 2 h after CIVAD (%)	32 (34.0)	30 (73.2)	19 (86.4)
Seizures within 2 h after CIVAD >50% PMD (%)	16 (17.0)	16 (39.0)	15 (68.2)
Breakthrough seizures (%)	35 (37.2)	33 (80.5)	21 (95.5)
Withdrawal seizures (%)	30 (31.9)	28 (68.3)	21 (95.5)
CIVADs changed (%)	20 (21.3)	20 (48.8)	17 (77.3)
Immune therapies (%)	25 (26.6)	11 (26.8)	6 (27.3)
Tracheal intubation (%)	51 (54.3)	30 (73.2)	20 (90.9)
Use of vasopressors (%)	32 (34.0)	19 (46.3)	15 (68.2)
Emergency resuscitation (%)	12 (12.8)	8 (19.5)	6 (27.3)

*CIVADs, continuous IV anesthetic drugs; PMD, proposed maximal dose; RSE, refractory status epilepticus; SE, status epilepticus; SRSE, super-refractory status epilepticus*.

* *Includes SRSE cases*.

*Data presented as n (%) or median (interquartile range)*.

### Responses to antiepileptic treatment

Thirty-two (34.0%) patients with SE due to acute encephalitis had seizures within 2 h after the initial use of CIVAD, and 16 (17.0%) patients still had seizures within 2 h after the rate of CIVAD was raised to >50% PMD. Breakthrough seizures occurred in 35 (37.2%) cases, and withdrawal seizures occurred in 30 (31.9) cases.

### Outcomes

**Figure 2:**
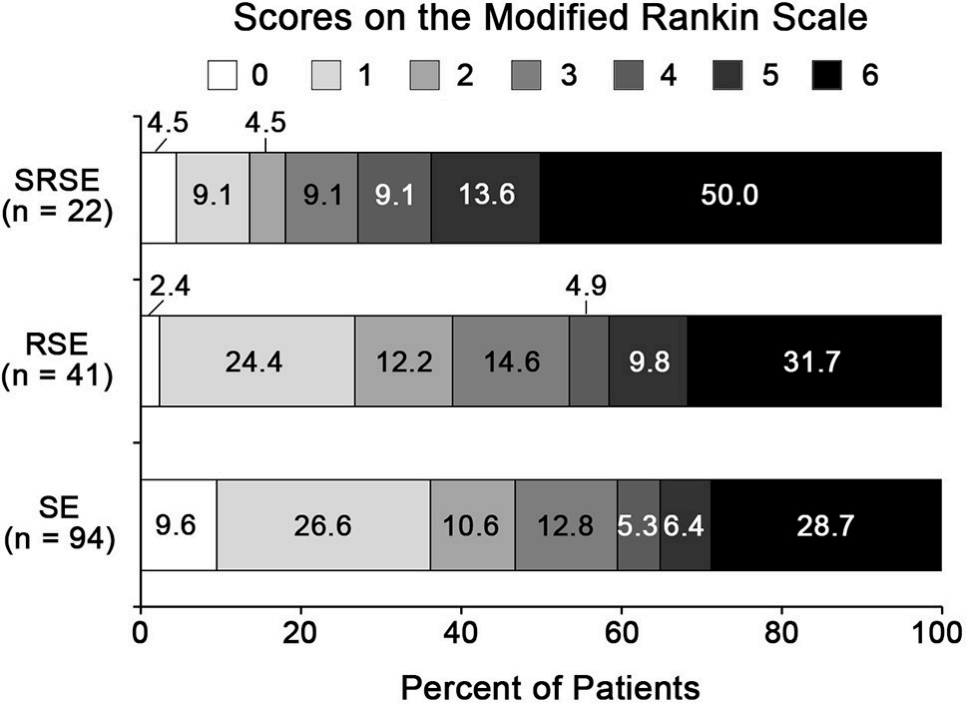
Three-month functional outcomes of SE in acute encephalitis. 0, no symptoms; 1, no significant disability; 2, slight disability; 3, moderate disability; 4, moderately severe disability; 5, severe disability; 6, dead. RSE, refractory status epilepticus; SE, status epilepticus; SRSE, super-refractory status epilepticus.

Forty-one (43.6%) cases of SE in acute encephalitis were refractory status epilepticus (RSE), 22 (23.4%) cases are SRSE. Thirty-eight (40.4%) patients with SE due to acute encephalitis had a poor outcome 3 months after N-ICU discharge, 19 (46.4%) RSE patients had a poor 3-month outcome, and 16 (72.7%) SRSE patients had a poor 3-month outcome (Figure 2).

### Prognostic factors for 3-month functional outcome

Table 4 showed that patients with a poor outcome had significantly less time from the onset of encephalitis to SE (*p* = 0.031), significantly higher STESS (*p* = 0.028) and END-IT scores (*p* = 0.001). Patients with a poor outcome were administered with significantly more IV AEDs (*p* = 0.031). Significantly more patients had NCSE in coma (*p* = 0.018), CIVAD >50% PMD (*p* = 0.042), seizures within 2 h after CIVAD >50% PMD (*p* < 0.001), CIVADs changed (*p* < 0.001), tracheal intubation (*p* < 0.001), the use of vasopressors (*p* < 0.001), emergency resuscitation (*p* < 0.001) in the poor outcome group. Multivariate logistic regression analysis identified that the recurrence of seizures within 2 h after CIVAD >50% PMD (*p* = 0.044), tracheal intubation (*p* = 0.011), and emergency resuscitation (*p* = 0.040) were independent risk factors for 3-month poor outcome (Table 5).

**Table 4:** Clinical characteristics of patients with favorable and unfavorable outcomes after status epilepticus associated with acute encephalitis.

	**mRS: 0–3 (*n* = 56)**	**mRS: 4–6 (*n* = 38)**	***P*-value**
Age, years	25 (18–39)	33 (18–45)	0.282
Male (%)	29 (51.8)	26 (68.4)	0.108
Time from onset to NICU admission, day	11 (7–23)	10 (6–21)	0.685
Time from onset to SE, day	7 (3–14)	5 (1–9)	0.031
Seizures before admission (%)	54 (96.4)	35 (92.1)	0.359
History of epilepsy (%)	5 (8.9)	3 (7.9)	0.860
GCS on admission	9 (6–12)	7 (4–12)	0.188
STESS	3 (2–3)	3 (3–4)	0.028
END-IT score	3 (3–4)	4 (3–5)	0.001
NCSE in coma (%)	26 (46.4)	27 (71.1)	0.018
Abnormal MRI findings (%)	32 (57.1)	28 (73.7)	0.101
Number of IV AEDs	2 (1–3)	3 (1–4)	0.031
Use of CIVADs (%)	24 (42.9)	21 (55.3)	0.237
CIVAD >50% PMD (%)	9 (16.1)	13 (34.2)	0.042
Seizures within 2 h after CIVAD (%)	16 (28.6)	16 (42.1)	0.174
Seizures within 2 h after CIVAD >50% PMD (%)	3 (5.4)	13 (34.2)	<0.001
Breakthrough seizures (%)	17 (30.4)	18 (47.4)	0.094
Withdrawal seizures (%)	14 (25.0)	16 (42.1)	0.081
CIVADs changed (%)	5 (8.9)	15 (39.5)	<0.001
Immune therapies (%)	16 (28.6)	9 (23.7)	0.599
Tracheal intubation (%)	20 (35.7)	31 (81.6)	<0.001
Use of vasopressors (%)	9 (16.1)	23 (60.5)	<0.001
Emergency resuscitation (%)	1 (1.8)	11 (28.9)	<0.001

*CIVADs, continuous IV anesthetic drugs; GCS, Glasgow coma scale; NCSE, nonconvulsive status epilepticus; NICU, neurological intensive care unit; PMD, proposed maximal dose; STESS, status epilepticus severity score*.

*Data presented as n (%) or median (interquartile range)*.

**Table 5:** Logistic regression analysis for three-month unfavorable outcome.

**Variables**	**Unadjusted analysis**			Adjusted analysis^*^		
	**OR**	**95% CI**	***p*-value**	**OR**	**95% CI**	***p*-value**
Age	1.02	0.99–1.04	0.230			
Male	2.02	0.85–4.78	0.110			
Time from onset to SE	0.99	0.97–1.01	0.412			
STESS	1.98	1.08–3.62	0.027			
END-IT score	1.94	1.31–2.87	0.001			
NCSE in coma	2.83	1.18–6.80	0.020			
CIVAD >50% PMD	2.72	1.02–7.23	0.045			
Number of IV AEDs	1.57	1.10–2.25	0.013			
Seizures within 2 h after CIVAD >50% PMD	9.19	2.40–35.17	0.001	4.52	1.04–19.68	0.044
CIVADs changed	6.65	2.16–20.50	0.001			
Tracheal intubation	7.97	2.98–21.36	<0.001	4.99	1.37–11.69	0.011
Use of vasopressors	8.01	3.05–21.02	<0.001			
Emergency resuscitation	22.41	2.75–182.67	0.004	9.80	1.11–86.47	0.040

* *Hosmer and Lemeshow Test: p = 0.970*.

## Discussion

In this study, we investigated the values of brain MRI and EEG monitoring for the predictions of progression to SRSE and 3-month functional outcome in SE due to acute encephalitis. Our data demonstrated that cortical or hippocampal abnormality on MRI and END-IT score independently predicted the progression to SRSE, and the recurrence of clinical or EEG seizures within 2 h after the infusion rate of a single anesthetic drug >50% PMD, the use of tracheal intubation, and the use of emergency resuscitation independently predicted 3-month poor outcome in patients with SE due to acute encephalitis.

SRSE is a life-threatening neurological emergency occurring in 4–16.9% of all cause SE (10, 32–37). The observed incidence rate of SRSE in our study of SE due to acute encephalitis was 23.4% which was much higher than the average incidence, and it was consistent with previous studies suggesting that encephalitis was the determinant of progression from SE to SRSE (10, 32). However, no particular etiology of encephalitis was found in our study to be associated with a higher incidence of SRSE.

Besides encephalitis, a lower premorbid mRS score and NCSE in coma were also indicated to be the independent predictors of SRSE (38). In our study, GCS, STESS, and END-IT score were chosen to assess the illness severity and investigated as the potential predictors of SRSE. GCS was initially designed to evaluate the level of consciousness. STESS includes consciousness, seizure type, age, and history of epilepsy. END-IT score encompasses etiology (encephalitis or not), NCSE, diazepam resistance, brain image, and use of tracheal intubation. The inclusion of measurements regarding more aspects of illness might be the reason why END-IT score was the independent predictor of SRSE in SE due to encephalitis.

Cortical regions and hippocampus have been demonstrated to be associated with epileptogenesis (39–46). In patients with acute encephalitis, cortical lesions on neuroimaging imply a high risk of early-onset status epilepticus (9). Our study also proved the predictive value of neuroimaging and found that the abnormality in cortex or hippocampus was an early predictor for the progression to SRSE in SE due to acute encephalitis. Further studies are needed to investigate whether a more aggressive anti-epileptic therapy will shorten the duration of SE and improve the outcome in those patients with a high risk of SRSE.

Compared to all cause RSE, patients with RSE due to acute encephalitis had higher rates of recurrent seizures within 2 h of the initial CIVAD treatment, breakthrough seizures, and withdrawal seizures (27). However, the recurrence of these seizures was not associated with a poor outcome. Only the recurrence of clinical or EEG seizures within 2 h after the initiation of a single CIVAD at a dose of more than half the proposed maximal dose predicted an unfavorable functional outcome at 3 months. The recurrent seizures under the CIVAD treatment are usually subtle or non-convulsive. Thus, continuous EEG monitoring not only plays an indispensable role in the monitoring and treatment of SE, but also contributes to the outcome prediction in SE due to acute encephalitis.

This study contained a larger sample size of SE due to acute encephalitis compared to previous studies, described the anti-epileptic treatment responses at length, and firstly identified early predictors of SRSE in SE due to acute encephalitis. However, this study had a retrospective observational design and was conducted in a single tertiary care center. Moreover, because our hospital is one of the largest hospitals in northwest China, many patients were referred from other hospitals. Some patients might have not received a timely and sufficient anti-epileptic treatment initially. In this study, we followed the Chinese guidelines on the management of SE (15), which were consistent with the European guidelines about the proposed maximal dose of CIVADs (14), but the maximal dose of midazolam we used was lower than the suggestions proposed in American guidelines (47). So far the treatment with high-dose midazolam for refractory SE has not been widely performed in China, future studies are needed to be conducted in China to investigate and validate the effects of different infusion doses of midazolam in SE patients.

## Conclusions

This study investigated the values of neuroimaging and continuous EEG in the multimodal predictions in SE due to acute encephalitis. Cortical or hippocampal abnormality on neuroimaging and END-IT score are independent predictors of SRSE. The recurrence of clinical or EEG seizures within 2 h after the infusion rate of a single CIVAD >50% proposed maximal dose predicts a poor outcome at 3 months after NICU discharge.

## Author contributions

FYu: Study concept and design, drafting of the manuscript, critical revision, statistical analysis, study supervision. FYa: Study concept and design, critical revision, study supervision. RJ, WL, YJ, JZ: Acquisition, analysis, interpretation of data. WJ: Study concept and design, critical revision, obtained funding.

### Conflict of interest statement

The authors declare that the research was conducted in the absence of any commercial or financial relationships that could be construed as a potential conflict of interest.
